# Examining non-technical skills for ad hoc resuscitation teams: a scoping review and taxonomy of team-related concepts

**DOI:** 10.1186/s13049-021-00980-5

**Published:** 2021-12-04

**Authors:** J. Colin Evans, M. Blair Evans, Meagan Slack, Michael Peddle, Lorelei Lingard

**Affiliations:** 1 Division of Emergency Medicine, Schulich School of Medicine and Dentistry, Western University, London, ON Canada; 2 Department of Psychology, Western University, London, ON Canada; 3 Middlesex-London Paramedic Service, London, ON Canada; 4 Centre for Education Research and Innovation, Schulich School of Medicine and Dentistry, Western University, London, ON Canada

**Keywords:** Resuscitation, Non-technical skills, Ad Hoc team, Scoping review, Prehospital, Emergency medicine, Trauma

## Abstract

**Background:**

Non-technical skills (NTS) concepts from high-risk industries such as aviation have been enthusiastically applied to medical teams for decades. Yet it remains unclear whether—and how—these concepts impact resuscitation team performance. In the context of ad hoc teams in prehospital, emergency department, and trauma domains, even less is known about their relevance and impact.

**Methods:**

This scoping review, guided by PRISMA-ScR and Arksey & O’Malley’s framework, included a systematic search across five databases, followed by article selection and extracting and synthesizing data. Articles were eligible for inclusion if they pertained to NTS for resuscitation teams performing in prehospital, emergency department, or trauma settings. Articles were subjected to descriptive analysis, coherence analysis, and citation network analysis.

**Results:**

Sixty-one articles were included. Descriptive analysis identified fourteen unique non-technical skills. Coherence analysis revealed inconsistencies in both definition and measurement of various NTS constructs, while citation network analysis suggests parallel, disconnected scholarly conversations that foster discordance in their operationalization across domains. To reconcile these inconsistencies, we offer a taxonomy of non-technical skills for ad hoc resuscitation teams.

**Conclusion:**

This scoping review presents a vigorous investigation into the literature pertaining to how NTS influence optimal resuscitation performance for ad hoc prehospital, emergency department, and trauma teams. Our proposed taxonomy offers a coherent foundation and shared vocabulary for future research and education efforts. Finally, we identify important limitations regarding the traditional measurement of NTS, which constrain our understanding of how and why these concepts support optimal performance in team resuscitation.

**Graphical abstract:**

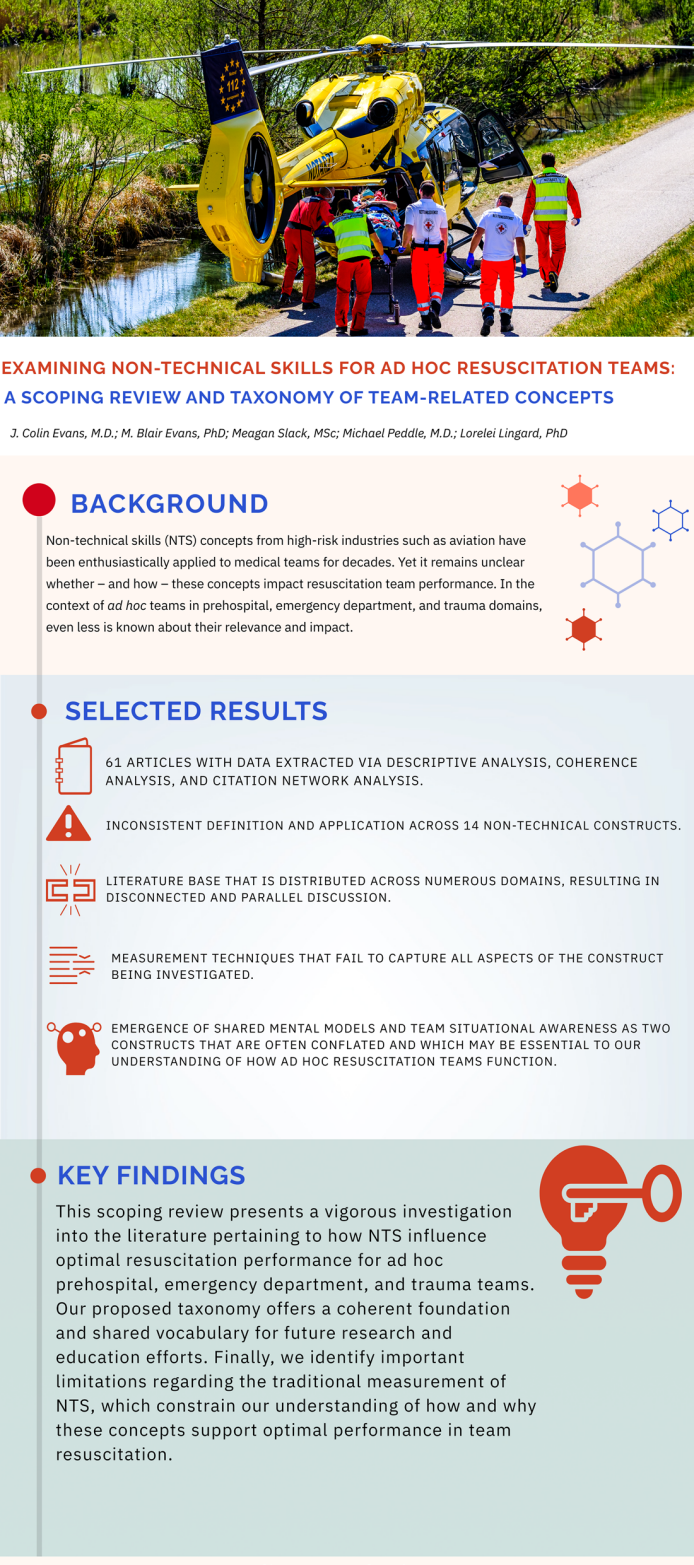

**Supplementary Information:**

The online version contains supplementary material available at 10.1186/s13049-021-00980-5.

## Introduction

Despite establishing the significance of teammate collaboration for resuscitation performance, resuscitation literature has yet to achieve a consensus regarding how non-technical skills (NTS) work and which constructs are most relevant to resuscitation teams. Interpersonal constructs like leadership, teamwork, and communication, and cognitive constructs such as decision-making and situational awareness have been studied in many settings and are now included within resuscitation guidelines around the world [1, 2]. Prehospital, emergency department, and trauma resuscitation teams perform in dynamic domains [3], experience frequent team membership turnover and integrate different professional cultures [4] all while expressing a high degree of interdependence [5]. The composition of these teams varies by region, but what these teams hold in common is their shared tasking as specialists in resuscitation and the necessity to unite members who are available to respond at the time of the patient’s critical event on an ad hoc basis. While there is now an extensive literature examining NTS for teams performing in these settings [6, 7], the specific impact of their ad hoc and intersectoral nature tends to be overlooked [8].

Ad hoc resuscitation teams, otherwise known as action teams [9, 10] and variable role, variable personnel (V_R_V_P_) teams [11], are composed in response to an acute demand for a limited performance [4] with variable membership including representation from various disciplines (e.g., emergency medicine, anaesthesia, surgery) and professions (e.g., physician, nurse, respiratory therapist). An added layer of complexity specific to prehospital resuscitation teams is their intersectoral nature: team members may also represent multiple sectors of society [12] (e.g., paramedic/EMT, physician, nurse, fire, police, lay responder), some of whom may have neither healthcare training nor a primary healthcare focus. Efforts to actively translate evidence from NTS literature into training and practice for resuscitation teams may be undermined if these findings are incompatible with the teams’ ad hoc dimension. A clear understanding of how NTS constructs relate to ad hoc teams is necessary to capitalize on – and meaningfully extend – the rich literature on NTS in resuscitation.

With this scoping review we take a configurative approach [13], which seeks to interpret and understand the state of resuscitation team literature. In contrast with an aggregative approach of combining empirical observations and making summative statements (e.g., meta-analysis), we used a configurative approach to identify key themes, clarify discrepancies, and describe gaps in the scholarly conversation pertaining to NTS for resuscitation teams. Through this lens, we classify each source based on team setting and structure, and the types of NTS constructs investigated—leveraging this review to interrogate existing theory and advance novel perspectives. Our aim is to provide future researchers and educators a clearer understanding of team dynamics and a common language for NTS, particularly as they pertain to ad hoc prehospital, emergency department, and trauma resuscitation teams.

## Methods

We selected scoping review as the most appropriate methodology to map the state of the literature pertaining to NTS in ad hoc team resuscitation. This approach allows us to describe the breadth of the literary landscape and account for its contours, unrestricted to methodology and setting or by a narrowly predefined research question [14]. Our search was guided by both PRISMA scoping review guidelines [15], and Arksey & O’Malley’s five step framework [14, 16] (see Table 1).

**Table 1:** Key phases in scoping review methods ( adapted from Arksey and O’Malley 2005)

Phase/stage	Goal of phase/stage
Stage 1	Identifying the research question
Stage 2	Identifying relevant studies
Stage 3	Study selection
Stage 4	Charting the data
Stage 5	Collating, summarizing and reporting the results

### Identifying the research question and search strategy

Leveraging the research question specified above, a preliminary list of keywords was first generated by brainstorming among members of the authorship team (which includes experts in medical teams and clinical aspects of resuscitation) regarding relevant terms and concepts. This keyword list was refined by reviewing concepts described in relevant studies using a database and google scholar search of the terms “non-technical skills” and “resuscitation”, and by cross-referencing all terms with the taxonomy applied to surgeons by Yule et al. [17]. This taxonomy was selected because our team regarded it as the most comprehensive and representative of the non-technical constructs identified in our preliminary search. This taxonomy distinguishes constructs as interpersonal skills (communication, leadership, teamwork, briefing/planning/preparation, resource management, seeking advice and feedback, coping with pressure/stress/fatigue) and cognitive skills (situation awareness, mental readiness, decision making, adaptive strategies/flexibility, workload distribution). With this keyword list, a research librarian assisted in selecting MeSH terms, database selection, and designing search queries. Our team chose to emphasize medical literature and selected four databases [EMBASE (Ovid), CINAHL (Ebsco), MEDLINE (Ovid), and Psychinfo (Ovid)]. The database search combined three groups of terms: 1. activity (e.g., resuscitation, ATLS, ACLS), 2. setting (e.g., prehospital, paramedic, emergency department), and 3. non-technical skills. An example of our CINAHL search query is available in online supplemental materials.

Study selection as well as inclusion and exclusion criteria were informed in an iterative fashion as our familiarity with the literature evolved. We primarily sought literature that specified a focus on prehospital or emergency deparment teams. Teams including other descriptions were included (e.g., trauma teams) in cases where our research team determined that teams described in the papers included emergency or prehospital members or when the clinical tasks took place in an emergency department context. Our final criteria are listed in the online supplemental materials and sought to identify manuscripts featuring original empirical studies as well as literature reviews that overtly measured or described NTS in the prehospital, emergency, and trauma settings. The inclusion of review articles in this dataset aligns with our configurative approach and speaks to our research question, which focuses on patterns regarding how relevant concepts were used in the literature.

### Data collection, charting and analysis

**Fig. 1:**
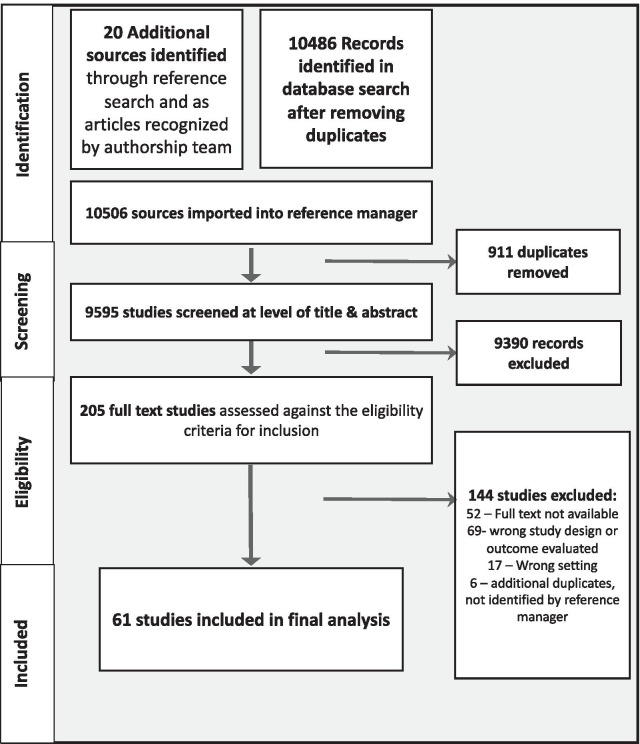
PRISMA flowchart

The PRISMA flowchart illustrating the progress of the search is available in Fig. 1. We performed our initial search in June, 2017 and a final update on October 12, 2021. The search identified numerous domains where resuscitation teams research was published and therefore supplemental search strategies (i.e., hand search of selected titles; grey literature search) were not integrated into this review. The database search results were combined with articles identified in our preliminary search and uploaded into the Covidence software platform [16] for duplicate removal, title & abstract screening, and full text eligibility screening. Two reviewers independently conducted title and abstract screening for all sources as well as subsequent screening for full text eligibility, with discrepancies resolved through discussion.

Our team performed three analyses of articles selected for data charting [14]: (a) traditional extraction of descriptive information, (b) coherence analysis to critically consider a study’s capacity to inform the literature, and (c) citation network analysis of articles included in this review.

Data extraction was performed by a single author using a Microsoft Excel (2018) spreadsheet. This analysis included categorizing broad themes (e.g., publication date, study type) as well as those more pertinent to our review (e.g., setting, team type, non-technical skills studied).

The heterogeneity of manuscript types and topics across the resulting articles in our dataset led us to employ ‘Coherence Analysis’ to explore how knowledge is being mobilized across this literature and situate each article by its influence on emerging theory. Traditional quality appraisals that entail a focus on methodological characteristics (e.g., risk of bias assessment) are ill-suited for scoping reviews. Instead, our coherence analysis aims to provide insight into how an article contributes to the scholarly conversation and uses an approach akin to those used by existing narrative reviews [17] and qualitative meta-syntheses [18, 19].

The coherence analysis involved three binary (Yes/No) items addressing: (1) Whether concepts related to non-technical skills team aspects were defined and operationalized (e.g., operational definition in main text), (2) Whether the article was situated within the broader literature by citing and appropriately characterizing relevant seminal works, and (3) Whether the article presents findings that contribute to our knowledge of non-technical skills. To assess intercoder reliability, our primary analyst and another author completed coherence analysis for ten articles. Across the 30 decision points, raters agreed on 26 decisions (87% agreement). The resulting Cohen’s Kappa value (*Κ* = 0.59; *CI* = 0.21–0.96) was acceptable.

Because the coherence analysis suggested that several articles were not well situated within the broader literature, we performed a citation network analysis to illustrate and explore relationships between articles. We examined the reference lists of included articles and cross-referencing citations for other articles in our dataset, producing a social network matrix identifying which articles were cited by those published later. The network matrix was visualized using Gephi software (v. 0.9.2), whereby the resulting network was descriptively analyzed alongside indicators of each article’s position within the network.

## Results

### Descriptive summary

The search query produced 9595 independent records screened by reviewers, from which 205 articles were reviewed at the level of full text. Sixty-one articles were included in the final analysis, which spanned 1992 to 2021 with forty-six (75%) articles involving original empirical data. Among the twenty (33%) intervention-based articles, six were randomized clinical trials or controlled experiments and fourteen described interventions delivered to a single group of participants (e.g., pre/post cohort study; descriptions of feasibility). Articles reporting on interventions used one of two approaches: either examining the implementation of specific policies or processes (e.g., team debriefing) or the implementation of targeted group interventions (e.g., TeamSTEPPS). Among the twenty-six nonintervention articles (43%), nine were qualitative articles involving interviews and/or observation, thirteen were quantitative articles using data drawn from clinical/training tasks (e.g., use of electronic health records, quantitative coding of video), two performed mixed methods analysis using both qualitative and quantitative assessments, and two were survey articles assessing staff perceptions of the salience of NTS. The analyzed articles also included thirteen (21%) narrative reviews and two comment articles.

In terms of the nature of the teams being investigated in these articles, thirty-eight (62%) articles referred to multidisciplinary teams with members from more than one discipline of medicine (e.g., surgical resident and emergency medicine resident), and forty-five (74%) had an interprofessional scope (e.g., physician, nurse, paramedic, respiratory therapist). With regard to our specific focus on ad hoc teams, twenty-eight (46%) articles addressed ad hoc team resuscitation, eleven (18%) articles examined prehospital responders, and five (8%) articles explored intersectoral teams (i.e., identifying members from multiple agencies such as paramedics and police) but none explicitly labelled these teams as intersectoral.

Among the non-technical skills for which we performed descriptive analysis, interpersonal skills were represented in fifty-eight articles (95%), while thirty-five (57%) explicitly examined cognitive skills. One observation from this review involves contrasting the attention directed toward interpersonal and cognitive skills over time. As evident in Table 2[4, 6, 8, 10, 20–76], interpersonal skills were the exclusive focus of analyzed articles until 2007—after which articles increasingly focused on both interpersonal and cognitive skills. This expansion of focus coincides with the 2006 release of the Yule et al. taxonomy [77]; however, our citation analysis found that only 5 (8%) articles [4, 46, 51, 62, 70] cited this taxonomy directly.

**Table 2:** Characterizing included articles regarding approach, coherence, and NTS included

Author, Year	Description [Coherence score]	Interpersonal Skills										Cognitive Skills				
		Communication	Leadership	Teamwork	Briefing & Planning	Resource Manage	Advice seeking	Stress or Fatigue Management	Followership	Debriefing	Decision-making		Situational Awareness	Mental Readiness	Adaptation	Shared Mental Model^1^
Driscoll & Vincent, 1992	Quantitative, observational [2]		*	*												
Xiao et al., 1996	Quantitative, observational [3]	*		*												
Stohler, 1998	Qualitative, interviews [2]	*		*												
Cooper & Wakelam, 1999	Quantitative, observational [3]	*	*													
Meerabeau, 1999	Review [3]	*	*	*												
Williams et al., 1999	Quantitative, observational [3]	*		*	*	*										*
Bergs et al., 2005	Quantitative and qualitative, observational [2]	*	*													
Cole & Crichton, 2006	Qualitative, interviews and observation [3]	*	*	*	*	*	*									
Hunt et al., 2007	Review [3]	*	*	*		*				*					*	
Campeau, 2008	Qualitative, interviews [2]	*	*	*		*					*		*		*	
Hicks et al., 2008	Quantitative survey, importance of NTS [3]	*	*	*		*					*		*			
Hoyer et al., 2009	Quantitative, observational [0]	*	*			*										
Manser, 2009	Review [3]	*	*	*						*			*		*	*
Andersen et al., 2010	Qualitative, interviews [3]	*	*			*										
Capella et al., 2010	Pre/post intervention, clinical setting [3]	*	*	*	*								*			
Hunziker et al., 2010	Controlled trial, simulation setting [2]		*													
Westli et al., 2010	Quantitative, observational [3]	*		*									*			*
Sarcevic et al., 2011	Qualitative, interview [3]		*	*							*					
Høyer et al., 2011	Controlled trial, clinical setting [2]	*	*	*	*	*										
Hunziker et al., 2011	Review [3]	*	*	*							*					
Steinemann et al., 2011	Pre/post intervention, clinical setting [1]	*	*	*		*		*			*		*			
Jankouskas et al., 2011	Pre/post intervention, simulation setting [0]			*		*							*			
Miller et al., 2011	Description of intervention delivery [0]	*	*	*									*			
Norris & Lockey, 2012	Review [3]	*	*	*				*	*	*			*	*		
Sarcevic et al., 2012	Qualitative, observational [3]	*	*	*							*		*			
Cooper et al., 2013	Review [3]												*			
Castelao et al., 2013	Review [3]	*	*	*	*											
Petrosniack & Hicks, 2013	Review [3]	*		*				*		*	*		*	*		*
Shields & Flin, 2013	Review [3]	*	*	*							*		*			
Clarke et al., 2014	Description of intervention delivery [1]	*	*	*	*	*					*					
Gjeraa et al., 2014	Review [3]	*	*	*				*			*		*			
Rasmussen et al., 2014	Quantitative survey, team experiences [3]	*		*												*
Clements et al., 2015	Pre/post intervention, clinical setting [2]	*	*								*		*			
Gillman et al., 2016	Description of intervention delivery [1]	*	*	*		*							*			
Lorello et al., 2016	Experimental design, simulation setting [3]	*	*	*										*		*
Maluso et al., 2016	Quantitative, observational [0]	*	*	*												
Holly et al., 2016	Review [3]	*	*	*	*	*	*	*			*		*		*	
Steinemann et al., 2016	Pre/post intervention, clinical setting [0]	*	*	*		*		*	*	*	*		*			*
Steinemann et al., 2017	Controlled trial, simulation setting [0]				*											*
Calder et al., 2017	Qualitative, interviews and observation [3]	*		*									*			*
Johnson et al., 2017	Quantitative, observational [3]	*	*										*			*
Myers et al., 2017	Experimental design, simulation setting [2]							*								
El Shafy et al., 2018	Quantitative, observational [3]	*	*													
Ghazali et al., 2018	Quantitative, observational [3]	*	*	*				*								
Hicks & Petrosoniak, 2018	Review [3]	*	*	*	*	*		*			*		*	*	*	*
O’Neill et al., 2018	Review [2]	*	*	*		*	*						*			*
Sullivan et al., 2018	Pre/post intervention, simulation setting [0]	*	*	*		*		*			*		*			
Herzberg et al., 2019	Quantitative, observational [1]	*	*	*		*					*		*			
Lazzara et al., 2019	Review [3]	*	*	*	*	*									*	
Murphy et al., 2019	Qualitative, interviews [3]	*	*	*					*				*			*
Coggins et al., 2020	Quantitative, observational and survey [3]									*						
Cormack et al., 2020	Review [1]	*	*	*		*					*		*			
Dagnell, 2020	Commentary [1]		*	*												
Dumas et al., 2020	Quantitative, observational [3]	*	*	*		*					*		*			
Fernandez et al., 2020	Controlled trial, simulation and live resuscitation [3]		*													
Gilmartin et al., 2020	Quality improvement report [3]									*						
Kristiansen et al., 2020	Pre/Post intervention, live resuscitation [0]	*	*	*												
Lapierre et al., 2020	Review [3]			*												
Sherman et al., 2020	Qualitative, observational [2]	*	*	*									*			
Armstrong et al., 2021	Pre/Post intervention, simulation [2]	*	*	*		*					*		*			
Petrosoniak et al., 2021	Quantitative, observational [3]	*	*	*									*			

### Taxonomy

Framed around Yule et al.’s NTS taxonomy for surgeons [77] and informed by our descriptive analysis, we created the Proposed Taxonomy of Non-Technical Skills and Team Constructs for Ad Hoc Team Resuscitation (Table 3) [4–7, 10, 20–42, 44–53, 57–66, 78–80]. This taxonomy represents our collective interpretation of definitions and applications presented in the literature integrated within this review, whereby we adapted the original Yule et al. taxonomy [17] and generated definitions regarding each construct that emerged from articles examining prehospital and ad hoc teams. As one key advance relative to the original taxonomy, the range of constructs has been broadened to include additional constructs identified in our review (i.e., debriefing, followership, and shared mental models). The novel taxonomy also identified a shift regarding the underpinning operationalization and classification of constructs. Whereas original perspectives of this taxonomy focused on *skills* with an ‘individual’ focus on training and preparation for individuals to contribute to teams, our revised taxonomy defines these constructs fundamentally as team processes (i.e., actions or behaviours observed when members combine their resources, knowledge, and skills as a team). Finally, the definitions and applications of these constructs that have emerged in this taxonomy confound classification as either interpersonal or cognitive and thus these categories have been removed.

**Table 3:** Proposed Taxonomy of Non-Technical Skills and Team Constructs for Ad Hoc Team Resuscitation

Nontechnical Skill/Construct Definition [f/% of studies from this review reporting on construct]	Considerations for Prehospital Ad Hoc Resuscitation Teams	Selected citations
**Leadership** The ability to develop team structure, maintain direction, and coordinate team activities. Leaders occupy a central role in facilitating communication with particular emphasis on developing and maintaining team situational awareness [47/77%]	Leadership is a subcategory of teamwork and central to teams’ success Members should maintain congruent and shared beliefs about which members are leaders Leadership may be fluid, transitioning between team members or distributed across numerous team members as task demands change or as team composition evolves Leaders often play a key role in team stress management, trust, and psychological safety, and maintaining a positive atmosphere Threats: 1.Ambiguous leader identity. 2. Leader performing technical interventions resulting in task fixation/loss of situational awareness	[4–6, 10, 20, 24, 28, 32, 34, 41, 44, 45, 51, 58, 65, 66]
**Communication** Verbal and non-verbal exchange of information. May occur within the team and between team and environment (e.g., patient, bystanders). Whereas communication may be task oriented or social in nature, communication is often studied in relation to the extent it develops situational awareness, performing mutual performance monitoring, and task delegation [49/80%]	Literature supports closed-loop communication (e.g., direction-verification-confirmation-acknowledgement, follow up once task complete) Leader communication is directed at maintaining situational awareness and task delegation, follower communication is directed at closing loop and volunteering information that will foster team situational awareness Communication effectiveness is increased when team member names are used and through direct delegation Graded assertiveness (e.g., Concern-Uncomfortable-Safety) model for patient safety is a means to overcome hierarchical barriers Threats: 1. Excessive/redundant information exchange. 2. Failure to use common language. 3. Cultural or hierarchical barriers resulting in indirect language and incomplete or inaccurate information sharing. 4. Environmental barriers and distractions	[5, 6, 20, 24, 28, 29, 32, 35, 42, 44–46, 50, 51, 57, 66, 75]
**Teamwork** A complex set of interactions amongst individuals who work adaptively and interdependently to achieve a common goal. Classically considered a broad construct and inclusive of other constructs such as leadership, followership, mutual performance monitoring, backup behaviour, adaptability, and team orientation [47/77%]	Patient safety literature in resuscitation has supported a transition from vertical integration, to a more horizontal structure that favours team input. Resuscitation teams feature characteristics of distributed cognition whereby working memory and pattern matching are greater than that of any individual member Resuscitation team composition is often fluid, with dynamic team member turnover, changes in or distributed leadership, and the presence of sub-teams with specialized tasks (e.g., airway team, compression team) Threats: 1. Ad hoc teams must unite under immense time pressure in conditions of significant complexity, as such team behaviours and structure are vulnerable to failure. 2. Vertical integration and hierarchy have oppressive impact on follower performance	[4–6, 29, 32, 34, 35, 42, 58, 65, 66]
**Briefing/Planning** Briefing: A targeted communication prior to commencing team resuscitation in which salient details of the impending resuscitation are delivered and a team plan of action and role assignment is determined via collaborative decision-making Planning: Updates occur throughout team activity via regular situation reports [10/16%]	Briefing is attributed to enhanced team mental models, decreased role ambiguity, and is associated with error reduction A four-step model is proposed to facilitate rapid prebriefing, these roles include: 1) what do we know?; 2) what do we expect? (plan A); 3) what will we change? (plan B); 4) role assignment Planning behaviours include updating of the initial briefing through regular “pauses” or “situation reports” and facilitates improved situational awareness and adaptive behaviours Threats: 1. Ad hoc resuscitation teams often assemble while the event is ongoing, eliminating the opportunity for prebriefing	[6, 28, 29, 36, 44, 56, 65]
**Resource Management** The assignment of team members and equipment to tasks in a way that is responsive to variable supply and demand of these assets. Requires the assessment of provider capacity/skill level and utilizing team members in such a way that optimizes their contribution. [21/34%]	Responsive to fluctuating resource support and demands (e.g., conflicting priorities, fatigue) Requires clear, direct, and specific task assignment Threats: Mass casualty response and prehospital resuscitation often modifies resource management requirements and situates individual patient resuscitation within a larger team environment. The construct applied to this circumstance is that of a multi-team system. In a multi-team system, team composition is highly reactive to fluctuating demands of broader incident priorities	[6, 20, 29, 31, 32, 51, 62, 65]
**Stress/Fatigue Management** Individual and team-based approaches to maintaining team performance by mitigating the adverse effects associated with stress and fatigue [11/18%]	Maladaptive stress response is associated with dysfunctions including degraded shared mental models, decreased performance in decision making, altered situational awareness, and impaired team function Fatigue is associated with deficits in resource management, teamwork, situational awareness, and decision making Leadership, mutual performance monitoring, backup behaviour, communication, are suggested as mechanisms to foster optimal team orientation, which can combat the detrimental effects of acute stress and fatigue	[6, 20, 41, 45, 51, 61]
**Followership** Who follows whom, the traits and characteristics exhibited by those in a following position, the process by which team members occupy a following role, and the influence that followers hold within the team [3/5%]	Refers to traits of resuscitation team members not assigned or fulfilling a leadership position. The corollary to leadership, an acknowledgement that most team members are not leaders but nevertheless exhibit characteristics that have the capacity to significantly impact team performance Followership research in resuscitation is limited, but there is increasing recognition that the earlier focus on hierarchical teams – with the resulting focus on leader behaviour – contrasts with recognition for the role of follower behaviours	[66, 87]
**Debriefing** A facilitated reflective process performed upon conclusion of team resuscitation efforts for the purpose of examining elements of optimal and suboptimal performance. [7/11%]	Debriefing allows opportunity for immediate feedback and fosters a culture of trust and support, facilitating improved team behaviours Threats: 1. The migration of the resuscitation environment from prehospital scene to emergency department functions as a barrier to effective whole-team debriefing. 2. Vertical team structure, lack of trust, and lack of psychological safety are all identified to negatively affect team member contribution to debriefing	[4, 29, 41, 45, 55, 68]
**Decision Making** A dynamic team process of interpreting data collected from the patient and environment to develop a working diagnosis and determine a course of action [19/31%]	Resuscitation decision making is often recognition-primed, with little space for deliberative decision making The concept of “distributed cognition” is proposed as a mechanism to describe team decision-making or “team mind” whereby the leader functions as the central executive and team members as evidence gatherers and treatment agents Studies identify that working memory and pattern matching are improved when decisions are made as a collective in resuscitation teams (e.g., collectives have a larger library of past experiences to contrast with the current situation)	[10, 42]
**Situational Awareness/Team Situational Awareness** Process of observing and interpreting ongoing clinical events and environments. Three steps: (1) perception of elements within a dynamic environment or system (e.g., patient); (2) comprehending the meaning associated with these observations; and (3) projecting these findings to support anticipation and response to future events. Evident at the individual level and at the team level [28/46%]	Situational awareness is foundational to decision-making and guides team coordination, communication, and behaviours Pre-briefings and intra-response situation reports (“Here’s what I see, this is what I think it means, and this is where I think we’re headed”) supports accurate team situational awareness and provides opportunity for correction Optimized by: (1) orientation at the beginning of the task; (2) maintenance during task and after disruption/change in environment; (3) recovery and reorientation after degradation Follower push communication (providing salient information without being asked) and communication of situational awareness is highly correlated with optimal team performance Threats: Task fixation, scene complexity, entrenched hierarchy/interagency silos, and stressful team environment can limit individual situational awareness while also limiting or eliminating voluntary team member contribution and thus diminish accuracy of team situational awareness	[6, 35, 46, 50, 51, 57, 58, 62, 66, 78, 80]
**Mental Readiness** Developing psychological skills for individuals and teams to regulate their mental state during performance. Attaining optimal arousal state during moments when demand nears or exceeds resources [3/5%]	Behaviours associated with the maintenance of an ideal performance state of arousal include: controlled or “tactical” breathing, self-talk, mental rehearsal, and activities that foster optimal team orientation such as prebriefings and maintaining mutual trust Stress inoculation training, mental practice, and overlearning are three training techniques that are associated with enhanced psychological skills for optimizing performance in acute stress environments An important foundational component of stress management	[6, 45, 53]
**Adaptive Behaviours** A team’s ability to anticipate and modify their structure and behaviours in response to dynamic changes in their patient’s clinical presentation and the environment. These behaviours are highly integrated with other NTS constructs including shared mental models, situational awareness, decision making, and debriefing [6/10%]	Based upon 4 adaptation phases: (1) situation assessment; (2) plan formulation; (3) plan execution; (4) team learning By virtue of understanding each team member’s role in relation to one another, teams with a strong team mental model have the greatest capacity for adaptive behaviour Optimizing team situational awareness through regular situation reports allows teams to more accurately and proactively predict dynamic changes resulting in more effective adaptive behaviours	[6, 20, 29, 51, 65]
**Shared Mental Model** Mental models reflect team members’ understanding of the team objectives, structures, and members’ roles within the team. Sharedness reflects the extent that members’ models are similar across the group [13/23%]	Shared mental models enable members to coordinate and anticipate one-another’s actions, even with limited discussion Other constructs from this review are known to support the strength of shared mental models, including orienting members as a team, backup behaviour between members, situational awareness, and effective leadership Associated with higher frequency of communicating and updating situational awareness Threats: Ad hoc and intersectoral teams may have a limited shared understanding of their team and tasks	[5, 6, 35, 45, 57, 58, 75, 79, 82]

### Coherence analysis

Through the coherence analysis, we identified that thirty-nine (64%) articles explicitly defined key terms. For example, in one article [10] the authors described contrasting leadership definitions based on the context of “stable teams” or “action teams”, which were characterized using references to describe both types of teams and leadership tasks associated with each. Twenty-two (34%) articles did not provide definitions for key terms and demonstrated inconsistency in their interpretation and application of key constructs. As an illustrative example, the concept of a shared/team mental model is one construct for which researchers held contrasting definitions and operationalizations.

The second coherence analysis component found that forty-six (75%) of the articles in our dataset were well-situated. In one article that achieved a “yes” rating, the authors used their introduction to extensively detail the history of non-technical skills rating systems [51]. We characterized the remaining fifteen articles (25%) as poorly situated because the articles did not introduce seminal works to situate key concepts, or because they misinterpreted the evidence base when situating their work.

The third coherence analysis component found that forty-nine articles (80%) used their discussion to contribute back to the understanding of how NTS influences team performance. For instance, a 2017 observational study by Calder et al. conducted interviews and in vivo observation to disentangle the conceptual overlaps in previous literature regarding team situational awareness, shared mental models, and team communication. In their discussion, they identified that their “findings contrast with previous work since we found that team members did in fact have a shared mental model” and that their work represented “the first comprehensive mixed method investigation of how inter-professional teams communicate during ED resuscitation” [57]. These findings contribute explicitly to the body of literature and move forward our understanding of resuscitation team performance. The twelve (20%) articles that received a “no” rating in this category failed to advance the scholarly conversation largely due to their presentation of non-specific claims that NTS interventions can improve team performance.

### Network analysis

**Fig. 2:**
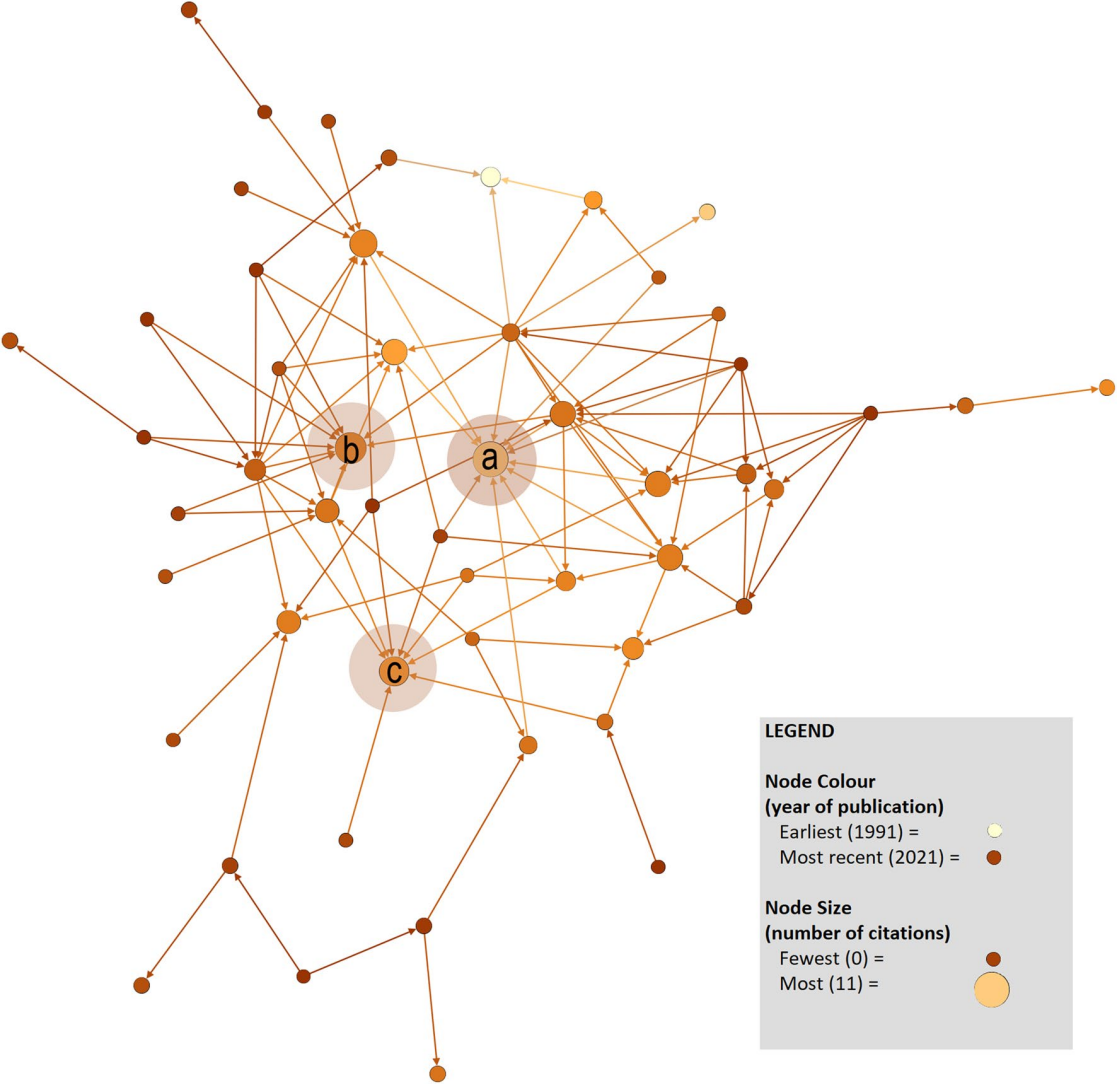
Citation network analysis. This network was created using Gephi and depicts the 61 articles in this review (nodes) and citations from a given source to another within this review represented by a directed arrow (ties). The size and orientation of each node was based on the number of citations an article received, whereas node colours distinguish articles by year of publication. Circles added to the figure denote papers with the highest number of citations, including: **a** Cooper (1994) (11 citations), **b** Capella and colleagues (2011) (9 citations), and **c** Hunt (2007) (7 citations). Note that this network figure only depicts 52 papers because nine papers included in this review had not cited other papers in this review, nor had they been cited by other papers in this review

Figure 2 is an illustration of the network comprised of articles included in this review. An arrow (tie) from one article to another reflects a citation. This network is limited to only the papers in this review, but it nevertheless characterizes the scholarly communities from which the field has emerged. The network was sparse, in that nine papers were both uncited and had not cited other papers in this review and only eight papers received more than five citations from others.

The network also provides an opportunity to reflect on the extent to which earlier publications received relatively more attention from subsequent articles in this domain: (a) Cooper’s reflection of leadership approaches in resuscitation [24] received 11 citations; (b) Capella and colleagues’ teamwork training evaluation with surgical residents [33] received nine citations, and (c) the review by Hunt et al. exploring simulation as a tool for enhancing team performance [29] received eight citations. Of particular interest within this network is the relative isolation of articles from outside of traditional clinical resuscitation outlets. Our figure highlights Sarcevic et al. [10], as a paper involving resuscitation teams that did not cite any earlier articles in this review and was cited only once by later articles in this review. Published in a medical informatics outlet that could have limited its exposure to scholars in other domains, this is one example of the challenge in how resuscitation teams research is dispersed across domains.

## Discussion

Our scoping review has identified the heterogenous nature of the disciplines, methodologies, and scope of articles pertaining to NTS for team resuscitation. While this diversity opens opportunities for growth and novelty, it also creates conditions for disconnected conversations that do not share a language and fail to accumulate into a refined model for how teams work during resuscitation. This discussion reflects on the nature and implications of such disconnected conversations within this field of inquiry. We also reflect on how our revised NTS taxonomy can redefine resuscitation teams research by facilitating consistent use of team-based concepts and by identifying emerging constructs that warrant exploration.

A key observation that has emerged from our coherence analysis and the supporting network analysis is that there are many disconnected, parallel scholarly conversations in the literature. Of particular note is the disconnect between articles published in clinical medicine journals and those published in non-medical domains such as human factors or applied ergonomics. Our coherence analysis revealed that specific non-technical skills were inconsistently defined across such domains, and the network analysis showed minimal cross-referencing occurring both within and between these two domains. These disconnects have profound implications for what we know about NTS for team resuscitation: insights already obtained in one field are ‘rediscovered’ in another; inconsistencies in terminology impede a cumulative refinement of knowledge; and the unique diversity of insight that might accompany interdisciplinary inquiry fails to materialize.

While NTS for individual practitioners [77] was the model around which this review was based and represents the conventional framing of this topic, the emerging discourse incorporates a wider spectrum of team processes. The Proposed Taxonomy of Non-Technical Skills and Team Constructs for Ad Hoc Team Resuscitation represents our effort mark this transition and bridge the disconnects that we identified within the literature base. Although scoping reviews are often used to aggregate and describe an evidence base, they are also a powerful tool to (re)configure the evidence base and advance theory [81]. Our taxonomy aims to identify and resolve inconsistencies in terminology that may limit future research and educational progress in this domain. It presents and defines NTS and team constructs that were targeted in studies within this field to-date, synthesizing definitions from the dominant approaches within the literature. Further, it includes examples of how these constructs have been applied in ad hoc teams and is informed by key insights from past empirical research.

This taxonomy could bridge the parallel discussions in this rich literature so that future scholars can contribute more coherently and purposefully to a shared knowledge base; however, the definitive nature of some constructs included in this taxonomy are limited by the quality and breadth of work to date. For instance, constructs of stress and fatigue management were included in our taxonomy because they were included in the initial taxonomy and reflected upon by 10 sources in our review but were often not positioned as a clear team process. Just as our review identified constructs like followership or shared mental models that weren’t integrated in earlier taxonomies, we present this as an evolving taxonomy with an expectation of future empirical investigation and refinement.

A particular area where the taxonomy can build coherence in the field relates to the popular constructs of shared mental models and team situational awareness. Whereas shared mental models refer to a situation in which “team members hold common or overlapping cognitive representations of task requirements, procedures, and role responsibilities” [79] pp. 222, situational awareness is “the perception of elements in the environment within a volume of time and space, the comprehension of their meaning and the projection of their status in the near future.” [81] pp. 36. Situationally-aware teams are those where members develop and maintain a collective understanding of a specific situation or patient presentation; as an acute ‘state’ of being situationally aware. Team members with shared mental models tend to enter a situation knowing their own (and others) roles as well as the goals of the group when they face given situations. Inconsistency in the use of these terms was a key finding of our coherence analysis. These two terms are often conflated across the studies [26, 56, 62, 66] or omitted insofar as findings allude to a construct while failing to explicitly reference it [23–25, 61].

An example of confounded definitions arises when articles indicate that situation reports develop a shared mental model. Whereas situation reports ‘can’ establish mental models when designed for this goal, the value of such reports is watered-down without considering how such reports also shape situational awareness and other group processes like leadership. The problem of omission is less conspicuous but arises when authors refer to generalized descriptions of effective teams as opposed to tangible and mutually exclusive concepts. For instance, one article argued that teams are optimal when they “have regular training, roles are well defined, and each can make safe assumptions about the level of preparation of others” [25] pp. 38. This claim lacks the precision that is gained when researchers use established concepts like shared mental models, role communication, or teamwork training. In contrast to the above examples, our dataset contains five recent articles wherein team situational awareness and team/shared mental models are described with the requisite nuance to capture their relationship [4, 6, 35, 57, 58]. These articles discuss these concepts as being essential for resuscitation team performance with one study finding that indicators of shared mental models explained as much as 23% of the variance in team performance outcomes [42].

It is critical for practitioners, researchers, and educators to distinguish between shared mental models and situational awareness because each involves differing challenges within ad hoc settings. Shared mental models are particularly elusive to promote in intersectoral prehospital ad hoc teams because they depend on entering situations with a collective understanding of how the team will ‘work’. Research is needed to examine whether strategies to promote shared mental models from other contexts (e.g., clinical leaders complete a training module on how to develop mental models) should be adapted in the context of ad hoc resuscitation teams. An additional area of focus lies in examining how these teams adapt in settings where a shared mental model does not exist or is not feasible. Ad hoc resuscitation teams clearly constitute a fertile setting to extend what we know about mental models and situational awareness from teams with more stable membership.

With improved clarity and consistency of the constructs associated with NTS in team resuscitation, we might also advance how we measure these constructs. While our descriptive analysis did not include a formal quality assessment, we observed that quantitative studies tended to examine key constructs by coding team interactions that could be observed during clinical experiences and simulations, or by intervening upon non-technical skills and measuring clinical outcomes like patient progress or procedural success. Measurement tools utilized in the studies included in our dataset focused almost exclusively on behavioral aspects of nontechnical skills while failing to evaluate the affective and cognitive components. This observation is mirrored in Cooper et al.’s systematic review examining measurement of situational awareness in emergency settings [48] as well as Lapierre et al.’s systematic review of studies examining simulation to improve trauma team performance [74]. These failings have also been identified in reviews involving other clinical contexts, which have recognized that studies examining teams rely on observational methods and are often inconsistent regarding how researchers define and measure group processes [83, 84]. The hazard in this approach is evident in the measurement of a team’s shared mental model through observation alone. Observation is a powerful tool for evaluating actions that might promote shared mental models (e.g., frequent communication) or observing the results (e.g., reduced conflict). Yet, observation is only a proxy for a team’s cognition. With observation alone we cannot directly estimate the extent that members share representations. In contrast, validated psychological measures of shared mental models often involve tools to identify critical aspects of teamwork in context, measure members’ individual perceptions of those aspects, and/or evaluate a group based on the degree to which members share representations [85]. Resuscitation researchers might adapt such tools to support both comprehensive evaluations of healthcare teams and specific measures of identified group processes and emergent states [86]. With valid measures for these constructs, we can delineate the nature of small group phenomena in resuscitation team performance and identify the active ingredients of interventions.

### Limitations

The selected databases focused on clinical medicine journals. While the few articles that we identified from non-clinical medicine journals have given us an indication of the divide that may exist between clinical and non-clinical journals, our search strategy did not capture the full breadth of investigations outside of the clinical medicine literature. Another limitation arises due to the inherent nature of the scoping review as an iterative process that redefines its inclusion and exclusion criteria as it traverses diverse territory. When applied to a heterogenous dataset such as this, the scoping review has the potential to leave those more accustomed to the rigid structure of systematic reviews and meta-analyses discomfited about what may have been left on the cutting room floor. Finally, citation network analysis constitutes an emerging analysis technique not usually included in scoping review methods. Constructing a network including only the studies from this review was useful to document how NTS definitions or measures have emerged within resuscitation literature; however, we did not document citations to sources outside of this review or external papers citing those included in this review. Researchers could use more comprehensive citation analyses to explore connections between resuscitation team literature and research from other clinical settings or areas of study.

## Conclusion

The literature on non-technical skills for ad hoc prehospital, emergency department, and trauma resuscitation teams is both diverse and disconnected. This review establishes that ad hoc resuscitation teams, and intersectoral ad hoc prehospital resuscitation teams, present realms that are ripe for future inquiry. We also offer a proposed taxonomy which presents a universal set of definitions for non-technical skills and team constructs for ad hoc resuscitation teams. We anticipate that this taxonomy will support the precision needed to incrementally advance understanding of teams in this context, such that insights obtained in one field can be applied in another, knowledge can accumulate across disciplines, and the rich insights of interdisciplinary inquiry can be realized. We also encourage future investigators to look beyond this literature base in search of validated psychological measures which more comprehensively assess the constructs being evaluated, so that the unique group processes responsible for collaboration in ad hoc teams can be more precisely described and enhanced through targeted training efforts.

## Supplementary Information

**Additional file 1.** **Supplemental Materials:** 1. Complete search query for CINAHL. 2. Inclusion Criteria Table. 3. PRISMA-SCR Checklist.

## Data Availability

Not applicable.

## Abbreviation

NTS: Non-technical skills
