# Supplementary material for: Examining non-technical skills for ad hoc resuscitation teams: a scoping review and taxonomy of team-related concepts
Source: Scand J Trauma Resusc Emerg Med. 2021 Dec 4;29:167. doi: 10.1186/s13049-021-00980-5 (PMC8642998; doi:10.1186/s13049-021-00980-5)
Supplement: Supplementary file 1 — Additional file 1. Supplemental Materials: 1. Complete search query for CINAHL. 2. Inclusion Criteria Table. 3. PRISMA-SCR Checklist. [file 13049_2021_980_MOESM1_ESM.pdf]

ONLINE SUPPLEMENTAL TABLES AND FIGURES

1. Complete search query applied to CINAHL
2. Inclusion criteria table
3. PRISMA-SCR Checklist

Appendix 1 Search String for CINAHL

S10 S4 AND S6 AND S9 Search modes -
Boolean/Phrase

S9 S7 OR S8 Search modes -
Boolean/Phrase

S8
Human Factor* OR non-technical skill* OR nontechnical skill* OR crisis resource management OR crew resource management OR CRM OR communication OR leader* OR awareness OR decision making OR interprofessional OR interprofessional OR interdisciplinary OR inter-disciplinary OR risk assessment OR group dynamics OR team* psychological adaptation OR mindfulness OR stress management judgement OR mindfulness OR cooperat*
Search modes -
Boolean/Phrase

S7
(MH "Decision Making, Clinical") OR (MH "Diagnostic Reasoning") OR (MH "Communication") OR (MH "Communication Skills") OR (MH "Communication Skills Training") OR (MH "Cooperative Behavior") OR (MH "Leadership") OR (MH "Teamwork") OR (MH "Perception") OR (MH "Executive Function") OR (MH "Mindfulness") OR OR (MH "Thinking+") OR (MH "Interprofessional Relations") OR (MH "Multidisciplinary Care Team") OR (MH "Consensus")
Search modes -
Boolean/Phrase

S6 S3 OR S5 Search modes -
Boolean/Phrase

S5 Ambulance OR prehospital OR Emergen*OR Rescue OR Paramedic* OR EMT OR first responder OR pre-hospital OR out of hospital
Search modes -
Boolean/Phrase

S4 S1 OR S2 Search modes -
Boolean/Phrase

S3 (MH "Emergency Medical Technicians") OR what about (MH "Rescue Work+")
Boolean/Phrase

S2 Resuscitat* OR advanced life support OR atls OR advanced trauma life support OR CPR OR ACLS
Search modes -
Boolean/Phrase

S1 (MH "Emergency Treatment+") OR (MH "Life Support Care+") Search modes - Boolean/Phrase

Online Supplemental Table.

Inclusion Criteria.

Domain	Inclusion criteria
**Publication date**	- Articles published after 1992.
**Language**	- Articles written in English.
**Publication type**	- Original academic journal articles.<br>- Empirical articles of any design (e.g., correlational, experimental, qualitative) or narrative reviews.<br><br>- Articles were excluded if they were not peer-reviewed and/or were considered grey literature.
**Constructs of interest**	- NTS constructs elated to the original Yule et al. taxonomy, which encompassed the following constructs:<br>o Communication, leadership, teamwork, briefing/planning, resource management, seeking advice, coping with stress, situation awareness, mental readiness, assessing risks, anticipating problems, decision-making, adaptive strategies, workload distribution.<br><br>- *What it meant to be a construct of interest was determined separately for empirical and review articles:*<br>o *Empirical articles that directly measured, intervened, or observed NTS.*<br>o *Narrative reviews whose purpose related to one or more NTS.*
**Clinical setting**	- Empirical articles with participants based within emergency departments, trauma teams, or prehospital care, or narrative reviews that describe application within these settings.
**Clinical activity**	- Participants of empirical articles were involved in activities that include resuscitation, either in training or clinical settings.<br>- Narrative reviews were included if they described applications toward resuscitation.

Note. The inclusion criteria requiring participants or discussions to involve resuscitation activities were removed for articles involving prehospital care. Specifically, we included four articles for this review that involved prehospital settings, and where related activities included (but were not limited to) resuscitation.

Preferred Reporting Items for Systematic reviews and Meta-Analyses extension for Scoping Reviews (PRISMA-ScR) Checklist

SECTION	ITEM	PRISMA-ScR CHECKLIST ITEM	REPORTED ON PAGE #
**TITLE**			
Title	1	Identify the report as a scoping review.	P 1
**ABSTRACT**			
Structured summary	2	Provide a structured summary that includes (as applicable): background, objectives, eligibility criteria, sources of evidence, charting methods, results, and conclusions that relate to the review questions and objectives.	P 2
**INTRODUCTION**			
Rationale	3	Describe the rationale for the review in the context of what is already known. Explain why the review questions/objectives lend themselves to a scoping review approach.	P 5
Objectives	4	Provide an explicit statement of the questions and objectives being addressed with reference to their key elements (e.g., population or participants, concepts, and context) or other relevant key elements used to conceptualize the review questions and/or objectives.	P 5
**METHODS**			
Protocol and registration	5	Indicate whether a review protocol exists; state if and where it can be accessed (e.g., a Web address); and if available, provide registration information, including the registration number.	Not included.
Eligibility criteria	6	Specify characteristics of the sources of evidence used as eligibility criteria (e.g., years considered, language, and publication status), and provide a rationale.	Online supplemental material
Information sources*	7	Describe all information sources in the search (e.g., databases with dates of coverage and contact with authors to identify additional sources), as well as the date the most recent search was executed.	P 6
Search	8	Present the full electronic search strategy for at least 1 database, including any limits used, such that it could be repeated.	Online supplemental materials
Selection of sources of evidence†	9	State the process for selecting sources of evidence (i.e., screening and eligibility) included in the scoping review.	P 6
Data charting process‡	10	Describe the methods of charting data from the included sources of evidence (e.g., calibrated forms or forms that have been tested by the team before their use, and whether data charting was done independently or in duplicate) and any processes for obtaining and confirming data from investigators.	P6
Data items	11	List and define all variables for which data were sought and any assumptions and simplifications made.	P6
Critical appraisal of individual sources of evidence§	12	If done, provide a rationale for conducting a critical appraisal of included sources of evidence; describe the methods used and how this information was used in any data synthesis (if appropriate).	P7
Synthesis of results	13	Describe the methods of handling and summarizing the data that were charted.	P7

SECTION	ITEM	PRISMA-ScR CHECKLIST ITEM	REPORTED ON PAGE #
**RESULTS**			
Selection of sources of evidence	14	Give numbers of sources of evidence screened, assessed for eligibility, and included in the review, with reasons for exclusions at each stage, ideally using a flow diagram.	Figure 1 (end of manuscript)
Characteristics of sources of evidence	15	For each source of evidence, present characteristics for which data were charted and provide the citations.	Table 2 (end of manuscript)
Critical appraisal within sources of evidence	16	If done, present data on critical appraisal of included sources of evidence (see item 12).	Table 2 (end of manuscript)
Results of individual sources of evidence	17	For each included source of evidence, present the relevant data that were charted that relate to the review questions and objectives.	Table 2 (end of manuscript)
Synthesis of results	18	Summarize and/or present the charting results as they relate to the review questions and objectives.	Table 3 and P8-
**DISCUSSION**			
Summary of evidence	19	Summarize the main results (including an overview of concepts, themes, and types of evidence available), link to the review questions and objectives, and consider the relevance to key groups.	P8-11
Limitations	20	Discuss the limitations of the scoping review process.	P16
Conclusions	21	Provide a general interpretation of the results with respect to the review questions and objectives, as well as potential implications and/or next steps.	P17
**FUNDING**			
Funding	22	Describe sources of funding for the included sources of evidence, as well as sources of funding for the scoping review. Describe the role of the funders of the scoping review.	Not funded.

JBI = Joanna Briggs Institute; PRISMA-ScR = Preferred Reporting Items for Systematic reviews and Meta-Analyses extension for Scoping Reviews.
* Where *sources of evidence* (see second footnote) are compiled from, such as bibliographic databases, social media platforms, and Web sites.
† A more inclusive/heterogeneous term used to account for the different types of evidence or data sources (e.g., quantitative and/or qualitative research, expert opinion, and policy documents) that may be eligible in a scoping review as opposed to only studies. This is not to be confused with *information sources* (see first footnote).
‡ The frameworks by Arksey and O'Malley (6) and Levac and colleagues (7) and the JBI guidance (4, 5) refer to the process of data extraction in a scoping review as data charting.
§ The process of systematically examining research evidence to assess its validity, results, and relevance before using it to inform a decision. This term is used for items 12 and 19 instead of "risk of bias" (which is more applicable to systematic reviews of interventions) to include and acknowledge the various sources of evidence that may be used in a scoping review (e.g., quantitative and/or qualitative research, expert opinion, and policy document).

From: Tricco AC, Lillie E, Zarin W, O'Brien KK, Colquhoun H, Levac D, et al. PRISMA Extension for Scoping Reviews (PRISMAScR): Checklist and Explanation. Ann Intern Med. 2018;169:467–473. doi: 10.7326/M18-0850.
